# Persistent Oxytetracycline Exposure Induces an Inflammatory Process That Improves Regenerative Capacity in Zebrafish Larvae

**DOI:** 10.1371/journal.pone.0036827

**Published:** 2012-05-10

**Authors:** Francisco Barros-Becker, Jaime Romero, Alvaro Pulgar, Carmen G. Feijóo

**Affiliations:** 1 Departamento de Ciencias Biologicas, Facultad de Ciencias Biologicas, Universidad Andres Bello, Santiago, Chile; 2 Instituto de Nutrición y Tecnología de los Alimentos, Universidad de Chile, Santiago, Chile; French National Centre for Scientific Research, France

## Abstract

**Background:**

The excessive use of antibiotics in aquaculture can adversely affect not only the environment, but also fish themselves. In this regard, there is evidence that some antibiotics can activate the immune system and reduce their effectiveness. None of those studies consider in detail the adverse inflammatory effect that the antibiotic remaining in the water may cause to the fish. In this work, we use the zebrafish to analyze quantitatively the effects of persistent exposure to oxytetracycline, the most common antibiotic used in fish farming.

**Methodology:**

We developed a quantitative assay in which we exposed zebrafish larvae to oxytetracycline for a period of 24 to 96 hrs. In order to determinate if the exposure causes any inflammation reaction, we evaluated neutrophils infiltration and quantified their total number analyzing the *Tg(mpx:GFP)^i114^* transgenic line by fluorescence stereoscope, microscope and flow cytometry respectively. On the other hand, we characterized the process at a molecular level by analyzing several immune markers (*il-1β*, *il-10*, *lysC*, *mpx*, *cyp1a*) at different time points by qPCR. Finally, we evaluated the influence of the inflammation triggered by oxytetracycline on the regeneration capacity in the lateral line.

**Conclusions:**

Our results suggest that after 48 hours of exposure, the oxytetracycline triggered a widespread inflammation process that persisted until 96 hours of exposure. Interestingly, larvae that developed an inflammation process showed an improved regeneration capacity in the mechanosensory system lateral line.

## Introduction

The aquaculture of fish, constitutes a rapidly world wide growing industry, especially for salmon and trout business. However this growth has been also accompanied by an increasing number of infectious diseases affecting fish, and therefore it has become very common to dose animals with antibiotics in the food to protect against illness [1]. As fish pens are typically located in rivers or lakes, feces, uneaten food pellets as well as antibiotic residues, are distributed over the entire ecosystem. Due to approximately 70 to 80% of the antibiotics administered are released into the aquatic environment, these chemicals must be considered potential environmental micropollutants [2]. Unfortunately, there are no reports about this problem and only limited information is available regarding the presence of antibiotics in the sediment surrounding the fish farms in a few countries [3]–[6].

The effects of antibiotics on the immune system in fish are numerous and can be different for each drug used. There is evidence suggesting that they can suppress immune functions in carp, rainbow trout, turbot and Atlantic cod [7]–[16]. However, the results reported from experimental investigations are contradictory as they depend on the type of assay developed and the fish species studied. Importantly, only the effect of antibiotics ingested or present in the bloodstream has been analyzed, but not the effect on the immune system triggered by antibiotics remaining in the water. As an alternative to analyzing the repercussions on a specific fish species and for unifying criteria, we decided to use the zebrafish (*Danio rerio*) as a model. This teleost fish has an especially well known biology, rapid development, is very easy to handle and allows us to make all the analysis *in vivo* and with a high number of specimens per data point [17]–[18]. Furthermore it has become of widespread use in ecotoxicology and toxicology research [19]–[22] and a particularly attractive and powerful new model for immunity research as it has number of strengths, including genetic tractability and transparency in embryonic and larval stage, which facilitates monitoring of infection processes [23]–[27].

In the zebrafish, the innate immune system becomes active early during somitogenesis, with fully functional macrophages appearing by 16 hpf and neutrophils at approximately 26 hpf [28]. In the case of an injury, both macrophages and neutrophils are able to migrate from the intermediate cell mass (ICM) (it will become the caudal hematopoietic tissue later during development) to the affected territories indicating that they are mature enough to fulfill a role as first defenders against an aggressor. Early during life of the fish, this innate immune system exists in isolation of an adaptive system, which only develops later in larval stages requiring 4–6 weeks to achieve a fully functional sate [29]. However, this adaptive immune system is very rudimentary in the zebrafish and consists mainly of B lymphocytes [30]. Therefore the gap between both systems allows considering infection of zebrafish larvae as a way to study exclusively the innate immune response without any adaptive immune contribution.

The hallmark of innate immunity response is inflammation. This process is triggered in response to injury, irritants, or pathogens [31]. If inflammation occurs there are influx, accumulation, and activation of leukocytes (predominantly neutrophils) at the site of injury during the early stages of the response [32]. These cells destroy the *injury agent* through the production of non-specific toxins, such as superoxide radicals, hypochlorite, and hydroxyl radicals [33]. Neutrophils also release several cytokines, including interleukin Il-1β, which is essential for inflammation progression. If the *injury agent* is removed, macrophage will secrete anti-inflammatory cytokines, such Il-10, which marks the end of the inflammatory process [34].

In the present study we investigate the effect of a prolonged exposure to oxytetracycline, one of the most commonly used antibiotics in finfish farming, on the innate immune response and regeneration capacity of zebrafish larvae. The goal is to establish an experimental model that allows us to analyze *in vivo* the effect on fish immunity of a persistent exposure to antibiotics, in many cases in high doses, present in the aquatic environment of fish farms.

## Results and Discussion

### Determination of LC_50_

Oxytetracycline is a broad-spectrum antibiotic with considerable activity against Gram-negative bacteria with worldwide use in fish farming [5]. The drug is administered to fish mixed in food at a dose rate of 50–100 mg per kg fish per day for 3 to 21 days. As during infection, fish usually show reduced feed intake and considering the low oxytetracycline bioavailability, it can be assumed that a considerable part of the medicated feed pass the treated fish uneaten and unabsorbed to the environment of the fish farm. Although, there is no current information about the concentrations of oxytetracycline detected in the water column or sediment in fish farms, only a few reports from the nineties are available and indicate that oxytetracycline is very persistent in fish farm sediments. In the environment surrounding pens up to 4.4 ug/g (4,4 ppm) of antibiotic has been detected even after 308 days of administration [35]–[37]. These levels rise dramatically in a freshwater recirculating system were after administrating the medicated feed for 10 days, the concentration in the sediment can reach 2150 µg/g (2150 ppm) [38]. This suggests that commercially relevant fish could be exposed to important doses of this compound after controlled administration.

Another important point related to the environmental oxytetracycline concentration is the amount of drug used, which differs greatly between countries. In the salmon industry, Norway and Chile are two world leader producers. During 2007 and 2008, to produce a tone of salmon Norway used 0,02 and 0,07 gr of antibiotic respectively. For the same period and production, Chile used 732 and 560 gr of antibiotic. This means that Chile used 36.600 and 8.000 times more antibiotics than in Norway [39] and therefore the fish farm environment is considerably more contaminated with these drugs.

Due to the lack of information about the concentration and effect of oxytetracycline in fish farms we decided to determine the LC_50_ at 24 hours post incubation (hpi), during embryonic development. The oxytetracycline used is a chloride form, which allows solubilization in aqueous media. Embryos at blastula stage were incubated in the antibiotic in 125 ppm, 250 ppm, 500 ppm, 750 ppm, 1000 ppm, and 1500 ppm and monitored every 6 hrs. At 6 hpi all the embryos at 1500 ppm and 1000 ppm were dead; later at 12 hpi, only a single embryo survived at 500 ppm. Finally at 24 hpi no more than two embryos survived at 125 ppm (Figure S1 A). Since it was not possible to determine the LC_50_ during embryonic stages, we decided to determine the highest sub-lethal oxytetracycline concentration (Oxy sub-lethal) in larval stages. To perform this experiment, we incubated 48hpf larvae in the same oxytetracycline dilutions used above for 4 days (Figure S1 B). On day 2 all larvae exposed to a concentration of 1500 ppm were dead; later at day 4, we find two (2/45) dead larvae at 1000 ppm. Due to we did not find larvae mortality with 750 ppm oxytetracycline in any of the 4 days studied, we decided to perform the next experiments using this concentration. To be sure that the amount of drug chosen remains sub-lethal after 4 days of incubation and that larvae will not die within a few hours after analyzed, we decided to incubate them 2 more days at 750 ppm. At day six of incubation all larvae were alive and we did not find any larvae with pericardial edema, abnormal heart function, delay in development or any other obvious detrimental phenotypic effect (Figure S2). These results indicate that the selected oxytetracycline working concentration is indeed sub-lethal, during at least 6 days of incubation.

### Persistent exposure to oxytetracycline induce neutrophils migration to superficial tissues of larval tail

**Figure 1:**
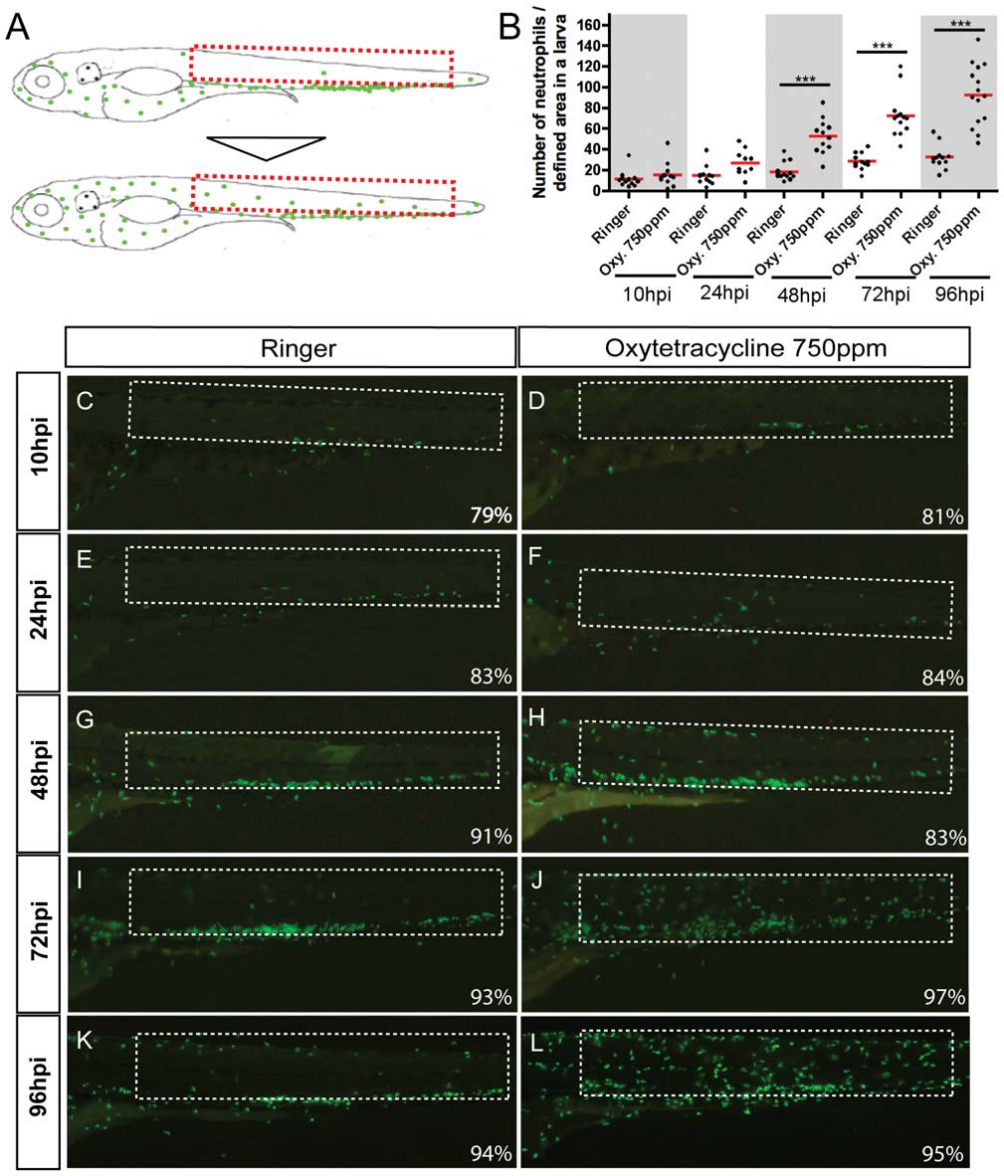
Exposure to oxytetracycline triggered an inflammation process. Incubation of *Tg(mpx:GFP)* transgenic larvae (that express GFP exclusively in neutrophils) in oxytetracycline 750 ppm induce a progressive migration of neutrophils from to the caudal hematopoietic tissue (CHT) to the entire tail. (A) Scheme of neutrophils localization in control and experimental larvae. (B) Quantification of neutrophils migration into the selected area. (C–L) Close up of the tail of control and experimental embryos at 10 hour post incubation (hpi), 24hpi, 48hpi, 72hpi and 96hpi. At 10hpi (C, D) and 24hpi (E, F) we did not detect any significant neutrophils migration. Later, at 48hpi (G, H) a discrete number of neutrophils migrate from de CHT to the tail. At 72hpi (I, J) and 96hpi (K, L), the presence of the GFP positive cells in the tail becomes clearly evident. The numbers of larvae that presented the phenotype shown is expressed as a percentage. For all experiments, at least 13 larvae were used for each condition. *** p<0.001.

As there is evidence suggesting that antibiotics can affect immune functions in other fish [7]–[16], we addresses the question if oxytetracycline exposure could modulate the innate immune function by triggering an inflammatory process. With this aim we decide to analyze neutrophils behavior in live zebrafish larvae. These types of leukocytes are the first cells to be mobilized in response to an injury and the first to infiltrate the damaged territory. To develop the assay we took advantage of the *Tg(mpx:GFP)^i114^* transgenic line, from now on *Tg(mpx:GFP)*, that expresses GFP under the control of the *myeloperoxidase* entire regulatory region and allows tracking individual immune cells in live animals [40]. We incubated *Tg(mpx:GFP)* larvae in 750 ppm oxytetracycline and monitored neutrophils migration at 10 hpi, 24 hpi, 48 hpi 72 hpi and 96 hpi utilizing a fluorescence stereoscope. To establish if indeed oxytetracycline induces neutrophils migration, we focused on the larval tail. In this region neutrophils are normally restricted to the caudal hematopoietic tissue (CHT) and only a few are circulating (Figure 1 A). It has been shown previously that tissue damage caused by injury, irritants, or pathogens, promotes neutrophils migration from the CHT to the site of damage [41] (Figure 1 A). Our results show that there is no significant recruitment of neutrophils in the tail at 10hpi nor at 24hpi (Figure 1 B–F). However at 48hpi we find a few neutrophils distributed throughout the experimental larvae tail, but by 72hpi an increasing number of neutrophils was evident in the analyzed territory (Figure 1 B, G–J). Finally, at 96hpi the migration becomes even more intense, indicating that we are clearly in presence of an inflammation reaction (Figure 1 K, L).

**Figure 2:**
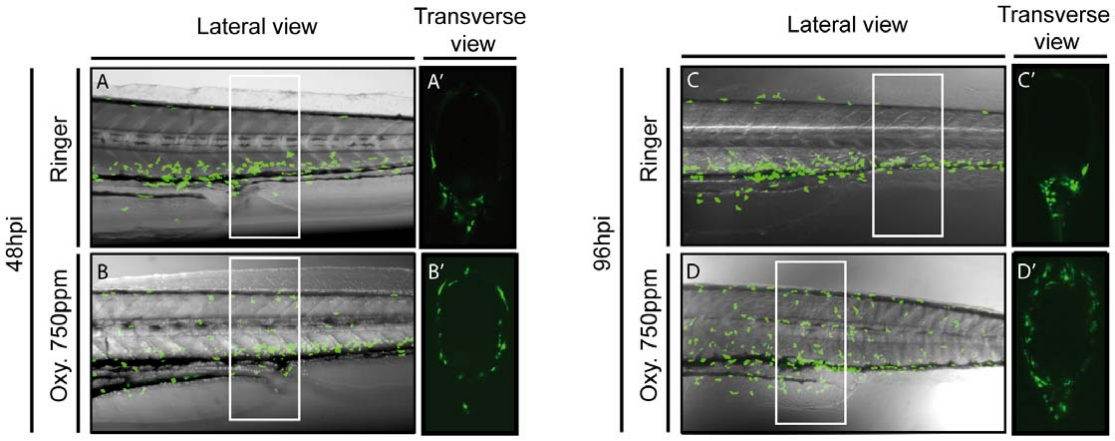
Oxytetracycline exposure induces neutrophils migration to superficial tissues. We analyzed the *Tg(mpx:GFP)* transgenic line, by confocal microscopy, detecting that in oxytetracycline exposure larvae neutrophils migrated to superficial tissue. (A–D) lateral view, (A′–D′) transverse view. Transverse views show a section of a larval tail of approximately 250 µm (white rectangle in lateral view).

To gain detailed insights into the inflammation process, we investigate neutrophils localization across the larval tail by using confocal microscopy. We thought that if the inflammation process is indeed triggered by the presence of oxytetracycline in the water and is at not a consequence of a general toxic effect, the inflammation must be more intense at superficial tissue (those that are in contact with the water such the mucosal epithelia) then internal organs, at least at early stages. We determined that in experimental larvae neutrophils were localized at superficial tissues both at the beginning of the inflammatory process (48hpi) and later (96hpi) (Figure 2 B′, D′), while in control larvae these polymorphonuclear cells remained throughout the ventral region, at the CHT (Figure 2 A′, C).

Together, our results suggest that waterborne exposure of larvae to oxytetracycline triggered a specific inflammatory response in external territories at larval tail. However at this point we cannot rule out that these observations could be due to massive cell death in treated larvae.

### Early stages of oxytetracycline induced inflammation are independent of cell death

**Figure 3:**
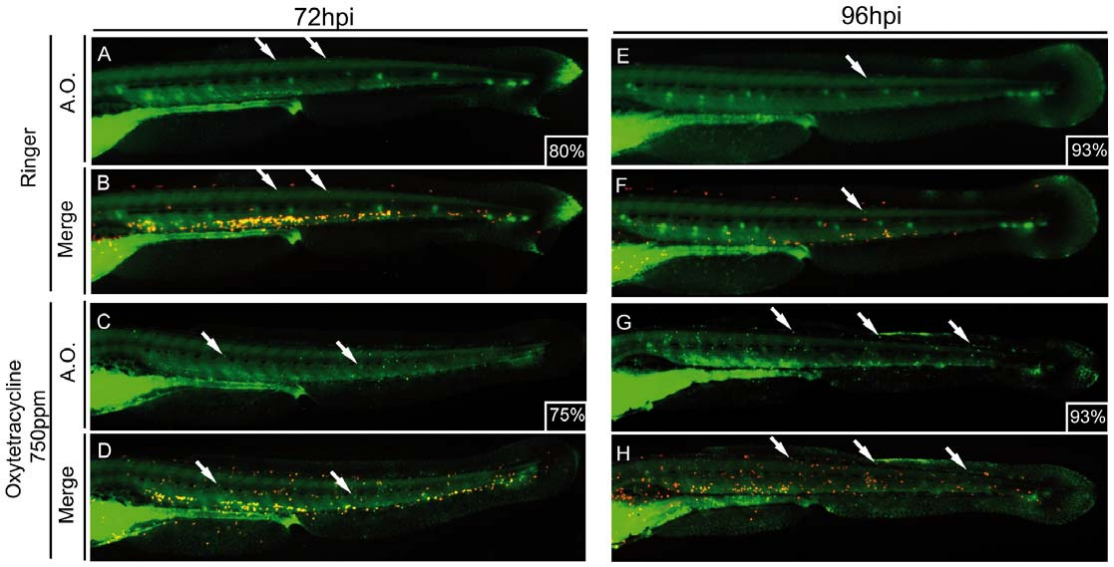
Cell death is detected in advanced steps of oxytetracycline induced inflammation. 48hpf *Tg(LyzC:DsRED2)* transgenic larvae were incubated for 72 hrs (A–D) or 96 hrs (E–H) in oxytetracycline 750 ppm followed by an acridine orange stain to address cell death. Experimental larvae showed higher level of cell death (white arrows) compared to control at both time analyzed. The numbers of larvae that presented the phenotype shown is expressed as a percentage.

Our previous results lead us to hypothesize that oxytetracycline triggers the inflammation process by inducing cell death in larvae. If we are right, we should observe leukocytes migrating to the areas where cells are dying. To explore this, we determined cell death levels by using acridine orange (AO), in transgenic larvae that express RFP in leukocytes *Tg(lyzC:DsRED2)nz50* [42], [43], herein named *Tg(LyzC:DsRED2)*. We found that there is no enhanced cell death in the whole embryo at 24hpi or 48hpi (data not shown) suggesting that the inflammation is not started due to massive apoptosis. Only at 72hpi, after neutrophils migration is initiated, a clear increase in the AO stain in the tail of larvae exposed to oxytetracycline was detected (Figure 3A–D); this was further intensified at 96hpi (Figure 3E, G). We analyzed if there was a correlation of this data and the leukocyte migration, but we found that these immune cells migrated in all directions, independently of the physical location of cell death (Figure 2F, H).

All together these results suggest that cell death was not the initial step of this inflammatory process. Although we cannot exclude that the observed cell death is a belatedly consequence of the direct action of oxytetracycline, a possible explanation to the late apoptosis, is that it could be a consequence of neutrophils action. These cells eliminate the invading agents by releasing toxic contents of their granules, but they do not discriminate between external and host targets. In this way, undesired damage is produced to host tissues [31].

**Figure 4:**
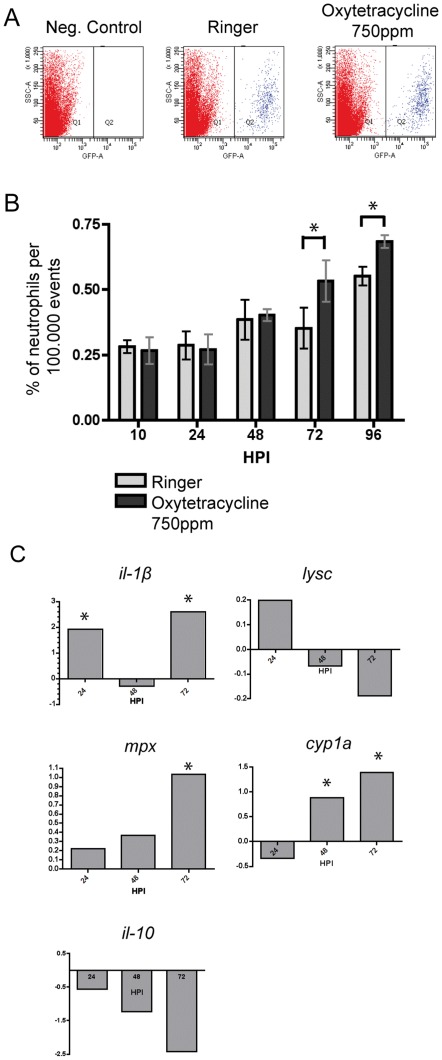
Neutrophils total number is increased in inflamed larvae. (A) To determine the total number of neutrophils a flow cytometry analysis was done. Three representative SSC v/s GFP graphics are showed. Cells from wild-type larvae were used to set the lowest level of GFP expression. (B) A significant increase in the number of total neutrophils is induced after 72 hrs of exposure to oxytetracycline. (C) Transcription levels of interleukin 1β (*il-1β*), interleukin 10 (*il-10*), mielloperoxidase (*mpx*), Lysozime C (*lysc*) and the cytochrome p450 1a (*cyp1a*) were quantified by qPCR. Transcript data were normalized to *β-actin1* and to the corresponding control. * p<0.05.

The ever-increasing number of neutrophils that infiltrate the larval tail under oxytetracycline exposure indicates that the inflammatory process becomes more intense and resolution is far from occurring, insinuating an increase in the total number of this type of granulocytes. To quantify the amount of neutrophils at every point analyzed before, we performed flow cytometry analysis (Figure 4A, B) using the *Tg(mpx:GFP)* line. The results obtained confirmed our previous observations, and a clear increase in the number of GFP positive cells was seen since 72hpi (Figure 4B).

**Figure 5:**
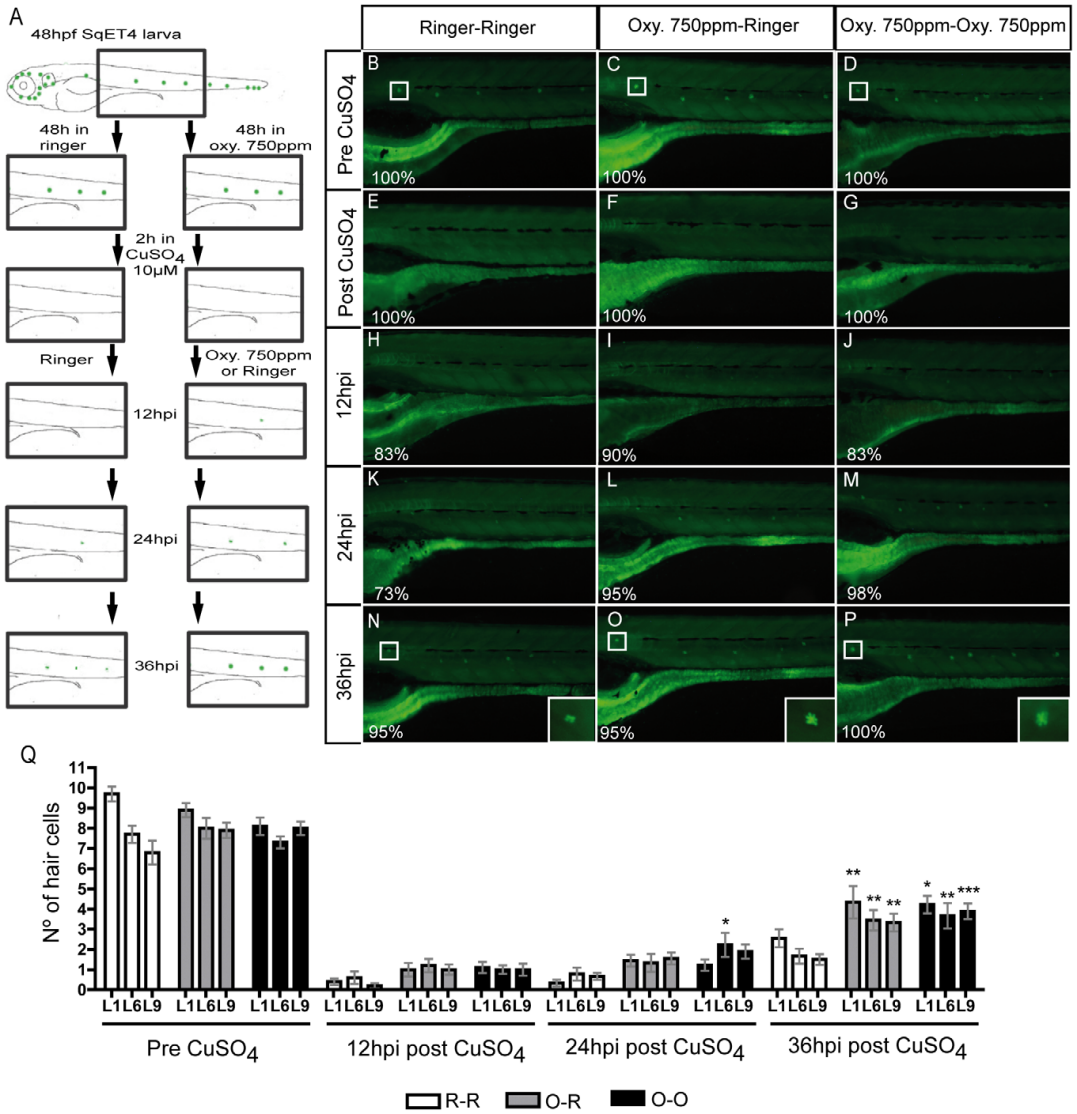
Oxytetracycline induced inflammation increase regeneration capacity. (A) Scheme of the regeneration assay developed. Incubation of SqET4 transgenic larvae (that express GFP in neuromast hair cells, HC) in oxytetracycline 750 ppm increases the rate of GFP/hair cells appearance. (B–D) HC before copper treatment. (E–G) CuSO_4_ treatment eliminates all GFP positive cells. (H–M, Q) Although we observe a tendency, there is no significant difference in the number of regenerated HC. (N–Q) At 36hpi the regeneration capacity of HC is significantly increased on oxytetracycline exposed larvae. (Q) The quantification of hair cells was performed in three neuromast; L1, L6 and L9. White box indicate L1 neuromast hair cells. The numbers of larvae that presented the phenotype shown is expressed as a percentage. * p<0.05, ** p<0.01, ***p<0.001.

**Table 1:** Primer sequences used for amplification of specific gene production with the RT-qPCR technique.

Gene	Forward Primer	Reverse Primer	Amplicon Size
***il-1β***	TGGACTTCGCAGCACAAAATG	GTTCACTTCACGCTCTTGGATG	150 bp
***il-10***	CACTGAACGAAAGTTTGCCTTAAC	TGGAAATGCATCTGGCTTTG	120 pb
***mpx***	TCCAAAGCTATGTGGGATGTGA	GTCGTCCGGCAAAACTGAA	90 bp
***lysc***	TGGGAGGCAATAGGCATGA	GCGTAGGATCCATCTGGTTTG	100 bp
***cyp1a***	GCATTACGATACGTTCGATAAGGAC	GCTCCGAATAGGTCATTGACGAT	120 bp
***β-actin1***	GCCAACAGAGAGAAGATGACACAG	CAGGAAGGAAGGCTGGAAGAG	110 pb

All together our results show that oxytetracycline promotes inflammation independently of cell death, at least at early stages of exposure, and it does induce an increase in the total amount of neutrophils in zebrafish larvae.

### Prolonged exposure to oxytetracycline induces expression of innate immune molecular markers

An early pivotal event in an inflammatory process is the increase in the transcription of pro-inflammatory cytokines, such Il-1β, enabling organisms to respond to different kinds of insults [31]. On the opposite side are the anti-inflammatory cytokines, such as Il-10, which are secreted mainly by macrophages when the inflammatory agent is removed, promoting the end of the inflammatory process [34].

As we mention before, other fundamental player of inflammation are neutrophils. These cells express myeloperoxidase (*mpx*), which is an enzyme stored in large amount in azurophilic granules [44]. Thus when new neutrophils differentiate, transcriptional level of *mpx* increase. On the other hand, when the inflammation occurs due to the presence of organic compounds, the transcription of the cytochrome *P*-450 monooxygenase enzymes is also induced, in particular the *cyp1a* subfamily. These enzymes are pivotal for the metabolism and biotransformation of many drugs [45]. In this circumstance, the nitric oxide generated by Mpx activity, can react with superoxide, produced by Cytochrome p450, generating the highly reactive peroxynitrite molecule, causing tissue damage [46].

In order to complement our previous results, we decided to monitor at molecular level the progression of the inflammatory process by evaluating several immune markers by qPCR. We evaluated well known immune markers at 24hpi, 48hpi and 72hpi (Figure 4C). At 24hpi we detected a significant increase in the transcript levels of *il-1β*, indicating that the inflammation process is already triggered at a molecular level. Later at 72hpi we identified a new peak of *il-1β*, event that support the increasing number of neutrophils outside the CHT. Likewise, a marked increase in the *mpx* transcription levels at the same time point was detected, result that also correlates with the enhanced total neutrophils number described before (Figure 4B). Finally, *il-10* levels were indistinguishable from control, at all the analyzed time points, which is consistent with our data showing an unresolved inflammation process (Figure 4B). Therefore, these data provides a quantitative support for our assay.

### Oxytetracycline induced inflammation increases regeneration capability

Due to an inflammatory process may alter the course of other physiological properties, we decided to evaluate changes in regeneration capacity in oxytetracycline treated animals. To assay regeneration capacity, we took advantage of a well-established protocol for monitoring effects of contaminants on the regeneration of mechanosensory hair cells in the fish posterior lateral line organ [41], [47]. The posterior lateral-line organ is a sensory system comprising a number of discrete sense organs, the neuromasts, distributed over fish tail in specific patterns. In turn, neuromasts comprise a core of mechanosensory hair cells, surrounded by support cells. Hair cells are a very good sensor of harmful compounds present in the water due to their localization on the surface of the fish [48], [49].

To perform the assay, we used the SqET4 transgenic line that specifically labels the mechanosensory hair cells (HC), allowing to monitor the presence of this cell type in live fish by detecting GFP expression [50]. With this aim we exposed 48hpf SqET4 larvae during 48 hrs to 750 ppm oxytetracycline (Figure 5A). Then, we incubated control and experimental larvae for 2 hrs with 10 µM CuSO_4_ to eliminate HC and washed with E3 medium to remove remaining traces of CuSO_4_ as reported before [41], [47]. At this point, we divided the experimental larvae into to groups, one incubated in ringer and other in oxytetracycline. This strategy allowed us to distinguish whether a possible effect in the regeneration is due to the antibiotic itself or to the inflammation triggered by it beforehand. After the copper treatment we monitored the appearance of HC in 3 specific neuromast, L1, L6 and L9, of the posterior lateral line in control larvae and the two experimental groups every 12 hrs. We choose those three neuromast since the position in the larval tail in which the new HC shall appear is easily identifiable. The obtained results show an evident acceleration in the onset of appearance of GFP positive cells in larvae treated with oxytetracycline after copper (Figure 5). Immediately after CuSO_4_ treatment, both experimental and control larvae loose all their hair cells (Figure 5 E–G) and although we observed a tendency, there is no significant difference in the number of HC at 12 and 24hpi (Figure 5 H–M). Later at 36hpt, neuromast in control larvae have a very low number of hair cells, between 1 and 3 (Figure 5N, Q). In turn, in both experimental assays L1, L6 and L9 have between 4 and 5 HC (Figure 5O–Q). This result indicates that it is not the antibiotic itself, but the inflammation that promotes the regeneration process. A similar effect of an inflammation process on regeneration was proposed by D'Alençon and collaborators [41]. A possible explanation is that in the inflammation process triggered by the exposure to oxytetracycline, a higher number of M2 macrophages are recruited to the damage zone compared to control larvae, thus increasing the regeneration capacity. Evidence in mammals indicates that when M2 macrophages are depleted after an injury, diminishing muscle repair, differentiation, and regeneration occur [51].

### Conclusion

In this paper we developed a battery of assays that allow, simply and rapidly, to evaluate aquatic environment impact on fish immune system. Our strategy considere the use of a number of biological markers of innate immune response that enables to determine if agents normally used or present in fish farms can induce these markers during or after exposure. Using a fish model increases the relevance of the study, as we are directly assaying bioavailable compounds with the target organism. Finally, it is of importance to establish which are the conditions that favor improvements in fish healthy and survival under conditions of stress, such as those found in salmon farms.

## Materials and Methods

### Ethics Statement

All animals subjected to experimentation were anesthetized and procedures complied with the guidelines of the Animal Ethics Committees of the Universidad Andres Bello, which approved this study.

### Zebrafish strains and maintenance

Zebrafish were maintained and raised in our facility according to standard protocols [52]. The following strains of fish were used in this study: Tab5 (wild type), *Tg(mpx:GFP)^i114^* [40], *Tg(lyzC:DsRED2)nz50* [43] and SqEt4 [50]. All embryos were collected by natural spawning, staged according to Kimmel *et al*. [18] and raised at 28,5°C in E3 medium (5 mM NaCl, 0.17 mM KCl, 0.33 mM CaCl2, 0.33 mM MgSO4, without methylene blue, equilibrated to pH 7.0) in Petri dishes, as described previously [53]. Embryonic and larval ages are expressed in hours post fertilization (hpf).

### Preparation of oxytetracycline solution and larvae Incubation

Six oxytetracycline (Zanil HCL 80%, Centrovet, Santiago, Chile) solutions at 125 ppm, 250 ppm, 500 ppm, 750 ppm, 1000 ppm and 1500 ppm were prepared in Ringer pH 7.0 medium (116 mM NaCl, 2,9 mM KCl, 1,8 mM CaCl_2_, 5 mM HEPES pH 7,2). The different solutions were stored in bottles protected from light and sealed with parafilm at 4°C for a maximum of 6 days. The use of a commercial oxytetracycline was validated by carrying out a set of incubations with pure oxytetracycline (Sigma). No differences between both antibiotics form at any concentration tested were identified. All embryos/larvae incubations were carried out basically in the same way. Briefly, 15 embryos, in the case of the LC_50_, or 15 larvae were transferred to six-well plate, in a volume of 5 ml of Ringer pH 7 until the experiments begun. At 3hpf or 48hpf respectively, the Ringer solution was replaced by the appropriated oxytetracycline solution. In the case of the LC_50_ assay, mortality was monitored every 6 hrs and for the determination of the heights sub-lethal oxytetracycline concentration (Oxy sub-lethal) every 24 hrs. All incubations were done in triplicate at a temperature of 28°C and at least three times. The time of incubation in oxytetracycline are expressed as hours post incubation (hpi).

### Acridine Orange Staining

For cell death characterization, zebrafish larvae were stained according to Williams *et al.* [42] with minor modifications. Embryos were incubated for 20 minutes in 5 µg/ml acridine orange (Sigma) in Ringer pH 7 medium, washed five times for 5 minutes in Ringer medium and observed under fluorescence stereoscope.

### Neutrophils migration and flow cytometry analysis

Neutrophils migration was addressed by analyzing, with a fluorescence stereoscope, the displacement of GFP positive cells in *Tg(mpx:GFP)* from the caudal hematopoietic tissue to the tail at 10hpi, 24hpi, 48hpi, 72hpi and 96hpi. For the flow cytometry, *Tg(mpx:GFP)* larvae at 10hpi, 24hpi, 48hpi, 72hpi and 96hpi (15 larvae per point in at least four independent experiment) were washed twice with PBS, followed by larvae disaggregation with 0.5% Trypsin-EDTA (Gibco) in PBS and resuspended in RPMI (Gibco) +1% FBS (Gibco) and passed through a filter with a 75 µm pore size and twice trough a 35 µm pore size. Cells from wild-type embryos were used to set the lower limit of GFP expression for each experiment. Flow cytometric analysis was performed on a FACScan (BD Biosciences).

### qPCR quantification

Larvae exposed to oxytetracycline 750 ppm since 48 hpf were sampled at 24, 48 and 72hpi for total RNA extraction. Control fish were sampled at identical time points. Samples included 25 larvae per time point per treatment. Total RNA extraction was obtained using Trizol Reagent (Invitrogen) according to the manufacturer's instructions. cDNAs were synthesized from RNA samples in a reverse transcription reaction using Super Script II RT (Invitrogen) according to the manufacturer's instructions and using oligo-dt primers.

Real time PCR was performed following descriptions of Rawls et. al. [54]. Each gene was tested in octuplicate and verified for non-specificity using melting curves (primers sequence in table 1). The mean Ct values from each sample were normalized against the mean Ct value of a reference gene (β-actin1, housekeeping gene). Relative quantification of each gene was obtained with the Pfaffl method and the REST 2009 software (Qiagen). This software includes a statistical test to determine accuracy of relative expression, which is complex because ratio distributions do not have a standard deviation. REST 2009 software overcomes this limitation by using simple statistical randomization test, the permuted expression data rather than the raw Ct values input by the user [55].

### Regeneration assay

Regeneration assay was carried out as described previously [47] with minor modifications. Briefly, fifteen 48hpf SqET4 larvae were incubated in Ringer pH 7,0 or oxytetracycline 750 ppm during 48 hrs. Then both control and experimental embryos were incubated in CuSO_4_ 10 µM (Merck) for 2 hrs to eliminate the hair cells, followed by 5 washes with E3 pH 7,0 to eliminate traces of CuSO_4_. Finally, control larvae were maintained in Ringer pH 7,0 and experimental larvae in oxytetracycline 750 ppm or Ringer during 36 hrs for monitoring hair cells regeneration and analyzed every 12 hrs.

### Imaging and statistics

Photographs were taken in an Olympus SZX16 stereoscope with a QImaging MicroPublisher 5.0 RVT camera and an Olympus BX61 microscope with a Leica DC300F camera. Confocal images were acquired with Olympus FluoView FV1000 Spectral Confocal Microscope (software version 2.1). Images were processed with Photoshop CS4 or Image J 1.44o. For all the experiments described, the images shown are representative of the effects observed in at least 70% of the individuals.

For statistical analysis Prism 4 (GraphPad Software) was used. The data was analyzed using one-way ANOVA for the neutrophils migration assay and two-way ANOVA for flow cytometry and hair cell regeneration assays.

## Supporting Information

Figure S1 **Determination of the oxytetracycline LC_50_ and maximum sub-lethal concentration.** Both, embryos (A) and larvae (B) were incubated in six different oxytetracycline concentrations ranging from 125 ppm to 1500 ppm and monitored every 6 hrs and 24 hrs respectively. The maximum sub-lethal concentration was the one where no mortality or any apparent phenotypic effect was detected. Results indicate that oxytetracycline was lethal to embryos in all the concentration analyzed. The highest sub-lethal concentration determined for larvae was 750 ppm.
(TIF)

Figure S2 **6 days of oxytetracycline exposure does not produce mortality nor adverse effects on larvae.** Larvae were incubated during 120 hrs and 144 hrs to ensure that the treatment with oxytetracycline does not produce any detrimental effects on larvae. We did not found phenotypic effects such cerebral edema, bending of the tail (A, D), pericardial edema, abnormal heart function (B, E), or any somite malformation (C, F). No change in survival rate was detected at the time analyzed (G).
(TIF)
