# Supplementary figures and images for: Persistent Oxytetracycline Exposure Induces an Inflammatory Process That Improves Regenerative Capacity in Zebrafish Larvae
Source: PLoS One. 2012 May 10;7(5):e36827. doi: 10.1371/journal.pone.0036827 (PMC3349639; doi:10.1371/journal.pone.0036827)

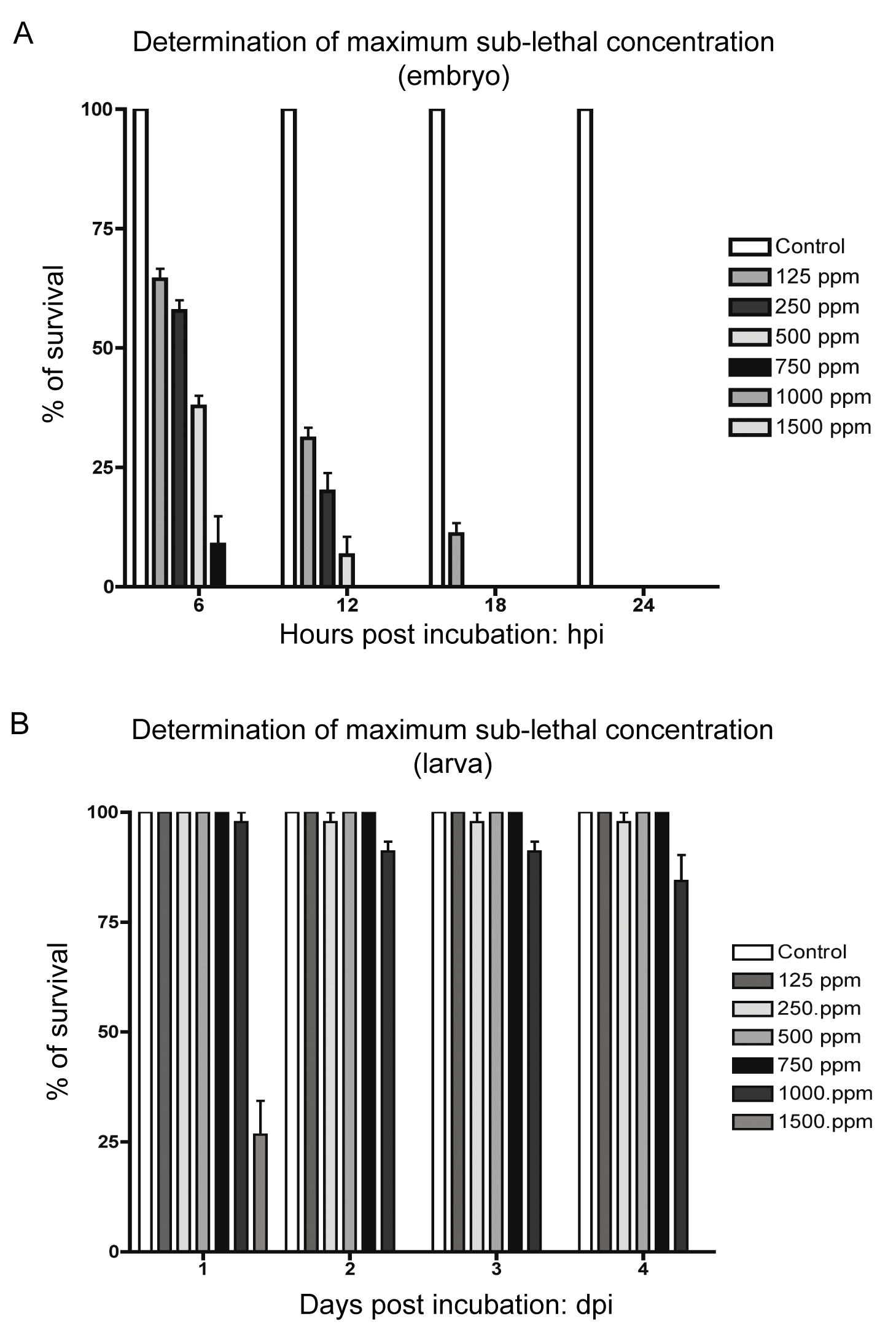

Supplement: Figure S1 — Determination of the oxytetracycline LC50 and maximum sub-lethal concentration. Both, embryos (A) and larvae (B) were incubated in six different oxytetracycline concentrations ranging from 125 ppm to 1500 ppm and monitored every 6 hrs and 24 hrs respectively. The maximum sub-lethal concentration was the one where no mortality or any apparent phenotypic effect was detected. Results indicate that oxytetracycline was lethal to embryos in all the concentration analyzed. The highest sub-lethal concentration determined for larvae was 750 ppm. (TIF) [file pone.0036827.s001.tif]

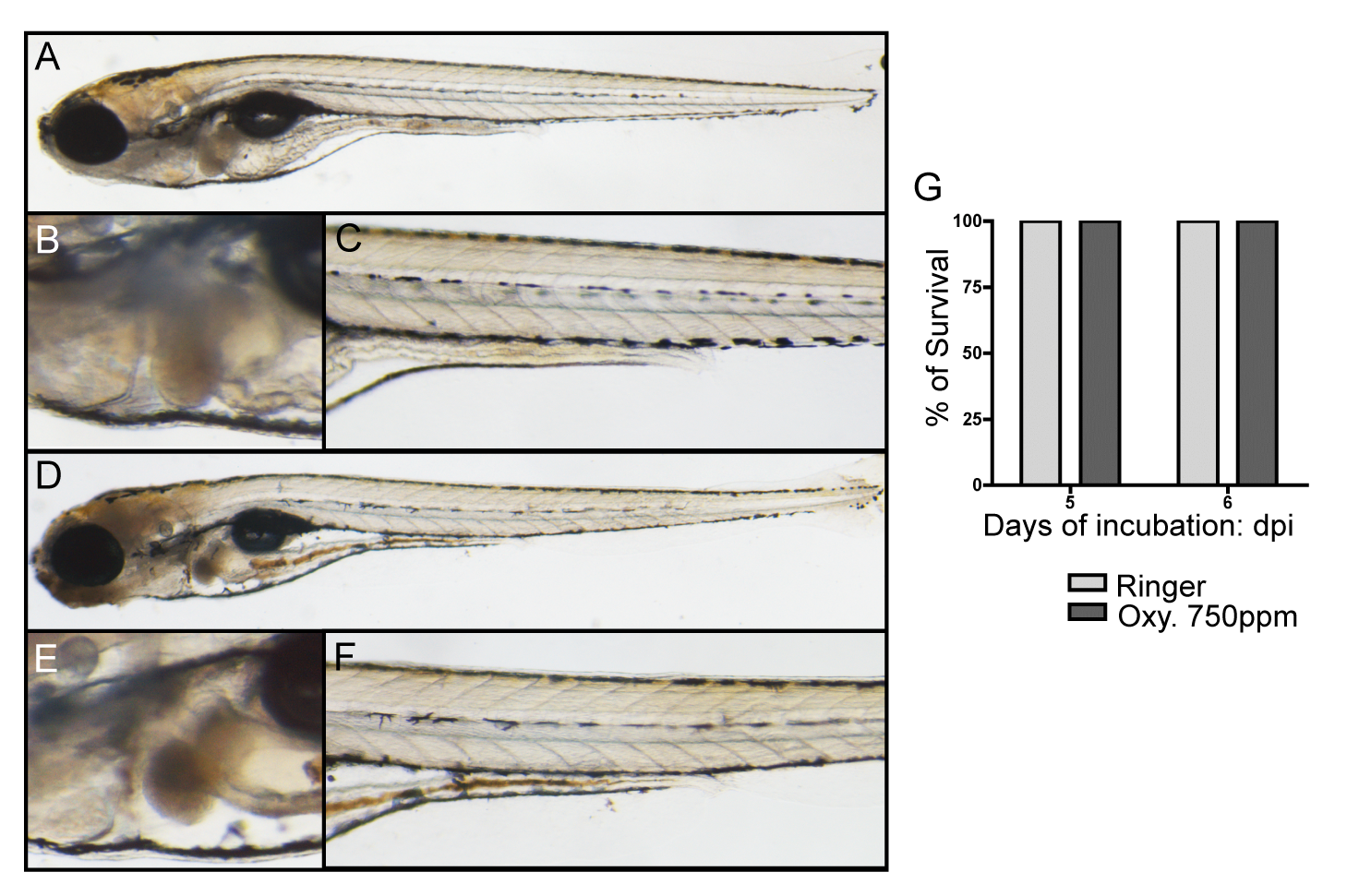

Supplement: Figure S2 — 6 days of oxytetracycline exposure does not produce mortality nor adverse effects on larvae. Larvae were incubated during 120 hrs and 144 hrs to ensure that the treatment with oxytetracycline does not produce any detrimental effects on larvae. We did not found phenotypic effects such cerebral edema, bending of the tail (A, D), pericardial edema, abnormal heart function (B, E), or any somite malformation (C, F). No change in survival rate was detected at the time analyzed (G). (TIF) [file pone.0036827.s002.tif]
